# Developing psychotherapists’ competence through clinical supervision: protocol for a qualitative study of supervisory dyads

**DOI:** 10.1186/1471-244X-13-12

**Published:** 2013-01-08

**Authors:** Margot J Schofield, Jan Grant

**Affiliations:** 1 School of Public Health and Human Biosciences, La Trobe University, Melbourne, VIC 3086, Australia; 2 School of Psychology and Speech Pathology, and Curtin Health Innovation Research Institute, Curtin University, Perth, WA, Australia

**Keywords:** Clinical supervision, Psychotherapy, Counselling, Mental health, Professional development, Professional wellbeing

## Abstract

**Background:**

Mental health professionals face unique demands and stressors in their work, resulting in high rates of burnout and distress. Clinical supervision is a widely adopted and valued mechanism of professional support, development, and accountability, despite the very limited evidence of specific impacts on therapist or client outcomes. The current study aims to address this by exploring how psychotherapists develop competence through clinical supervision and what impact this has on the supervisees’ practice and their clients’ outcomes. This paper provides a rationale for the study and describes the protocol for an in-depth qualitative study of supervisory dyads, highlighting how it addresses gaps in the literature.

**Methods/Design:**

The study of 16–20 supervisor-supervisee dyads uses a qualitative mixed method design, with two phases. In phase one, supervisors who are nominated as expert by their peers are interviewed about their supervision practice. In phase two, supervisors record a supervision session with a consenting supervisee; interpersonal process recall interviews are conducted separately with supervisor and supervisee to reflect in depth on the teaching and learning processes occurring. All interviews will be transcribed, coded and analysed to identify the processes that build competence, using a modified form of Consensual Qualitative Research (CQR) strategies. Using a theory-building case study method, data from both phases of the study will be integrated to develop a model describing the processes that build competence and support wellbeing in practising psychotherapists, reflecting the accumulated wisdom of the expert supervisors.

**Discussion:**

The study addresses past study limitations by examining expert supervisors and their supervisory interactions, by reflecting on actual supervision sessions, and by using dyadic analysis of the supervisory pairs. The study findings will inform the development of future supervision training and practice and identify fruitful avenues for future research.

## Background

Mental health professionals face unique demands and stressors in their work, resulting in high rates of burnout and distress [1-3]. There have been growing calls to address these stressors through professional support and development mechanisms [4-7]. Clinical supervision, in particular, is regarded as a key method for helping to avoid burnout and maintain professional wellbeing [7,8] as well as for holding mental health professionals accountable [8-10]. Clinical supervision refers to a principled and contractual relationship, in which the supervisor assists the supervisee to reflect on their clinical work with patients/clients [11]. Depending on the purpose and current need, supervision is likely to focus on: skills training; theorising and case conceptualisation; clinical, professional, and ethical reflection; mentoring; professional development; and personal support [8,9,12-14].

Clinical supervision has been widely adopted as an essential part of the training experience in psychiatry [15], psychology [5,8,14,16], and other mental health professions [13]. Psychotherapists, in particular, view ongoing clinical supervision as the key method for improving professional competence [17], and undertake supervision long after professional requirements are met [18,19]. While it has long been advocated as part of ongoing professional development [8,9,12,14], it has more recently become a mandated career-long requirement for both psychiatry and psychology in Australia [20,21]. When undertaken by more experienced therapists, it is often referred to as ‘peer supervision, ‘peer consultation’, or ‘peer review’ [20,21].

Despite widespread endorsement of clinical supervision and the development of a growing number of theoretical models of supervision [9,10,12,14,22], the evidence base underpinning the practice of supervision is very limited. In particular, there is limited evidence about the effective components of good supervision [15,23], and we know even less about its impact on client outcomes [23]. However, research does demonstrate that bad supervisory experiences have a negative impact on supervisees. For instance, an Australian survey on psychiatry trainees indicated that those who were disappointed with their supervisory relationship did not cope well with adverse client related events [24,25], and reported high levels of despair and psychological problems [25]. Such findings are important because they suggest that negative supervision experiences may result in supervisees avoiding the input of their supervisors [26], even when it is critical for effective practice, or for therapist or client wellbeing [24].

Theoretical writers agree on the importance of the quality of the supervisory relationship as a core ingredient in supporting and facilitating professional development [9,11]. Furthermore, a comprehensive review of the psychotherapy field has highlighted the need to understand the essential characteristics of empirically supported relationships, as a complement to the focus on investigating empirically supported treatments [27]. An emerging body of research on the nature of the supervisor-supervisee relationship suggests that problematic supervision is characterised by confrontational criticism, the direct attribution of blame, unclear agendas, and instructive, rather than interactive learning processes [28]. Since supervisory relationships are often long-term and have the potential to impact on lifelong careers, and potentially thousands of clients treated over that time, it is essential that we gain a more in-depth understanding of the processes involved and how to improve the effectiveness of supervision. To capture important processes of the supervisory alliance, research is needed that includes both supervisor and supervisee perspectives and explores how they work together to construct an understanding of the therapeutic process, and importantly, how supervisors influence changing perceptions and behaviours in supervisees.

The mental health practice field has become increasingly complex since deinstitutionalisation [29]. Along with this, the ethical, boundary, and systemic challenges have increased, heightening the need for development and evaluation of robust clinical supervision methods [29]. The field has been challenged to gather more evidence on the core competencies of supervision [16], and on the development of effective supervisors [8,14]. In addition, there have been calls for the practice of supervision to be better informed by adult learning methods [4].

The current study responds to these calls for a stronger evidence base to underpin supervision practice by examining the clinical supervision of psychotherapy practice by highly experienced supervisors with their experienced supervisees. The focus is on how supervisor-supervisee dyads co-construct understandings of the supervisees’ therapeutic challenges and how this impacts on supervisees themselves, their way of working in therapy, and on perceived client outcomes. It explores supervisor and supervisee conceptualizations, actual supervision practice, including the here-and-now supervisor-supervisee relationship, the process of learning taking place, and supervisor-supervisee reflections on the impact of supervision on client outcomes.

The study addresses past gaps in the research literature given that few studies have examined the supervision process from the perspective of highly experienced or expert supervisors, or from the supervisor-supervisee dyadic perspective, and very few studies have used recorded supervision sessions as a source of data, instead relying on retrospective report of events [23]. The study, therefore, has potential to contribute research-based insights into effective supervision processes that enhance competence in supervisees and how this impacts on their work with clients and client outcomes.

The specific aims of the study are to:

1. Explore expert clinical supervisors’ theories, practices and experiences of supervision, to better understand the processes that build supervisee competence.

2. Examine actual sessions of expert supervisor and experienced supervisee dyads to increase understanding of the supervision processes underpinning the development of competence in supervisees.

3. Examine expert supervisor and supervisee dyads’ views on the ways that supervision can improve client outcomes.

4. Synthesize findings into a model of optimal supervisory practice that links supervision practices with supervisee performance and impact on client outcomes.

Table 1 provides further details on the aims of the study and the key features of the study protocol in relation to these aims.

**Table 1:** Research aims, intended methods of addressing these, and potential benefits from this line of enquiry

**Research Aim**	**Methods**	**Significance**
1. To examine expert clinical supervisors’ theories, practices and experiences of supervision, and better understand the processes that build supervisee competence.	In-depth interviews with 16 highly experienced clinical supervisors who were nominated by peers as expert supervisors. Topics are shown in Table 2.	Most research has examined supervision of trainees rather than experienced therapists, and the supervisors of trainees rather than exploring the clinical wisdom of highly experienced or expert supervisors. This research fills these gaps in literature.
2. To examine actual sessions of expert supervisor and experienced supervisee dyads to increase understanding of the supervision processes underpinning the development of competence in supervisees.	Separate in-depth (interpersonal process recall) interviews with supervisor-supervisee dyads while watching DVD recorded supervision session to better understand how supervisors go about the task of building competence in supervisees and supervisee experiences of this process (see Table 3 for sample questions).	To contribute to the literature by examining the actual practice of supervision through DVD recordings; by analyzing both supervisor and supervisee views and experiences of joint interactions; and undertaking dyadic analysis. The results will inform supervisor training and professional development programs, and yield a richer picture of supervisory processes.
3. To examine expert supervisor and supervisee dyads’ views on the ways that supervision can improve client outcomes.	Separate in-depth (interpersonal process recall) interviews with supervisor-supervisee dyads while watching DVD recorded supervision session to better understand how supervision may impact on client outcomes (see Table 3 for sample questions).	Very little research evidence exists on whether supervision improves client outcomes and the processes by which this happens. This study will increase the evidence base about how supervision influences actual client outcomes, with implications for the funding and mandating of supervision.
4. To synthesize findings into a model of optimal supervisory practice that links supervision practices with supervisee performance and impact on client outcomes.	Following full analysis of the qualitative data from both supervisors and supervisees, and their dyadic analyses, a model will be constructed and tested from the data.	The rich body of data will provide a deeper understanding, not only about the key components of supervision, but also about the process of implementing different supervisory practices and techniques, and how supervisors move from one to the next. A fuller picture will be presented of the complexity of clinical supervision and how expert supervisors integrate the theory, context, supervisee and client characteristics into effective supervisory practice.

## Methods and Design

Clinical supervision practice requires the application and refinement of theory to the unique contexts, stories, and presentations of supervisees, consistent with definitions of evidence-based practice [30]. However, the predominance of quantitative research on supervision does not yield a sufficiently detailed, nuanced, or rich description of the highly complex learning processes that occur within the supervisory dyad [23,31]. In order to delineate the supervisory processes that build professional competence this study utilises a mixed method qualitative research design, allowing for differing perspectives (supervisor, supervisee, and DVD recorded session data). It also aims to deepen reflective activity through multiple interviews, reflection on actual sessions, and the analysis of dyadic as well as individual perspectives.

### Conceptual framework

The methodological framework of the study is guided by three key influences: social constructivism [32], phenomenology [33], and the reflective practitioner model [34]. Using a social constructionist lens, supervision is understood to involve reflective, inter-subjective processes involving co-construction of meaning [32]. The method of enquiry must, therefore, be a dynamic process of exploring the multiple layers of supervision, therapy, and client, and one that allows for the elaboration of both inner reflective activity and clarification of understanding developed through dialogue.

The second methodology guiding the study, phenomenology [33], is particularly suitable for the study of supervision processes because of its emphasis on the experience of participants. As a methodology, it allows us to examine not only what is said, but also what is experienced subjectively within the participants, and how this impacts on outer dialogue and action. In our study, phenomenology is further informed by a hermeneutic perspective, providing an interpretive lens [35]. Hermeneutics has been described as the process of uncovering hidden meanings [35].

The third influence comes from Schön’s reflective practitioner model [34], and its recognition that much professional knowledge emerges from experience, rather than theory. Espoused theory is our conscious view of what informs our practice, and what is likely to be accessed in a traditional interview method. However, what is espoused may not be what actually informs our practice. Theory-in-use, on the other hand, is the theory that actually directs practice (implicit theory). One purpose of supervision is to facilitate the supervisee to engage in the meta-cognitive process of reflecting on their practice, in light of theory and the broader context. In Schön’s terms, this *reflection-on-action* is the process of making sense of an action after it has occurred, leading to hidden or tacit knowing, and resulting in a more integrated understanding. Schön also describes the meta-cognitive process of *reflection-in-action*, the capacity to reflect ‘on the spot’ in the midst of professional activity. An important function of supervision (*reflection-on-action*) is to enable practitioners to learn how to incorporate these reflective practices into their everyday working expertise (*reflection-in-action*).

### Design

Research that seeks to understand the meta-cognitive process of *reflection-on-action* (clinical supervision), and how, in turn, supervision improves the therapist’s capacity for *reflection-in-action* (therapy) must utilize methods that capture the complexity of these processes. Thus we adopted a mixed method qualitative approach that involves two phases.

#### Phase 1

In the first phase, an in-depth face-to-face interview is undertaken with the purposively selected sample of expert clinical supervisors. A semi-structured interview guide is used to elicit open-ended reflections on their approach to supervision, their experience of helpful and unhelpful supervision, good and poor supervisory relationships, their understanding of how supervision builds competence in supervisees and better outcomes for their clients, and factors that influence supervision effectiveness. Core interview questions are shown in Table 2. Responses to the core questions can be elaborated using further prompts and follow-up questions.

**Table 2:** Interview schedule for expert supervisor interviews

**Interview topic**	**Key interview questions**
Supervision approach	· Tell me about your actual practice of supervision. What does a typical supervision session look like? Does this vary with different supervisees? · What are the skills and knowledge you feel are most essential for supervisees to develop? What emphasis do you place on conceptual or personal development of supervisees? How do you do that? · How does your method, model or theory of supervision differ if it is for formal requirements (eg registration, membership of an association, linked to a training)
Supervision theory	· Do you have a theory or model that underpins your supervision work? Can you explain how you use this and how you may adapt it to the supervisee? · How has your model developed – what have been the key influences on its development?
Supervision techniques	· Do you use specific techniques in supervision? Do you have a predominant focus in your sessions – eg feelings, cognitions, countertransference, alliance, skills? · How do you go about assessing and planning your work with supervisees?
Essence of good supervision	· What do you think are the key qualities/characteristics of good supervision - the essence of good supervision? · What are the skills and knowledge you feel are most essential for supervisees to develop? What emphasis do you place on conceptual or personal development of supervisees? How do you do that? · Can you describe a particularly good supervision session – what made it a good session?
Difficulties in supervision	· Can you describe a session that wasn’t effective? What made it not effective? · Can you describe a time when there was a major problem in the supervisory relationship – how did you deal with this? · In general, how would you raise and explore relationship difficulties in a session? Can you give an example? · What are the characteristics of supervisees that you think you are most/least effective with? Can you explain why? Are there specific examples you can think of? · What sort of supervisory events do you find most difficult to deal with – can you give examples? Do you use specific theories to help you understand what might be happening when things are difficult? Do you share these theories with your supervisee?
Person of the supervisor	· What do you think supervisees value most about you as a supervisor? How might they describe you as a supervisor? · How does the person you are impact on the supervision you provide? What do you think supervisees value most about you as a supervisor? How do you think they might describe you as a supervisor?

#### Phase 2

Following the first interview, supervisors are asked to assist with the recruitment of one of their current supervisees and record a supervision session on DVD. Separate interviews with the supervisor and their supervisee are then undertaken while reviewing the DVD recording. These interviews use social constructionist and phenomenological lenses to explore reflections on the observed supervision session. Specifically, the Interpersonal Process Recall (IPR) interview method [36] is used to foster a reflective practitioner stance [34] and seeks to understand, in detail, the moment-by-moment thinking, action, and experience of the supervisor and supervisee in interaction with each other (while watching the recorded session). The IPR interview method has the potential to activate deeper exploration of the learning processes by opening up a space to understand more fully the complex set of factors influencing each of the supervisors’ and supervisees’ understandings and actions. The researchers are engaged in a process of eliciting and interpreting the supervisors’ and supervisees’ expert knowledge, using social constructivist notions that knowledge is constructed and refined through dialogue. Participants are regarded as the experts on their experience of supervision, how it assists supervisees to develop competence, and how it influences their practice and client outcomes. The IPR methodology allows us to: anchor the data collected in Phase 1 to specific supervisory experiences from the DVD; elicit the thoughts and feelings of participants in the session; *reflect-on-practice*; and explore how it may influence future practice.

### Sampling and recruitment

#### Supervisor sampling

A purposive sample of 16 to 20 highly experienced supervisors is being identified and recruited by the researchers through professional networks in Australia and the UK. Colleagues, such as senior academics, senior practitioners, departmental heads, and heads of institutes and colleges, are asked to nominate supervisors they would consider to be expert supervisors. The aim of this purposive sampling criterion is to maximise the level of supervisor competence and to yield rich data from the accumulated clinical wisdom of senior supervisors. It privileges supervisor expertise over sample size or representativeness. Eligible supervisors are then sent written information and consent forms inviting them to participate and are followed up by phone.

#### Supervisee sampling

Each consenting supervisor is asked to recruit one of their supervisees by providing written information about the study. Willing supervisees are asked to contact the research team directly, to avoid any pressure on the supervisory relationship. Supervisees must be willing to participate in an in-depth interview, allow a supervisory session to be DVD recorded, and engage in an IPR interview where they would reflect on the session and their overall supervision experience. Supervisees are assured that a decision not to participate will not affect their rights to supervision. While it is recognised that supervisors may recommend only supervisees with whom they have a good relationship, this is not considered a major threat to validity, since past research demonstrates discrepancies between supervisor and supervisee perceptions of the supervisory alliance [37], as has been found for perceptions of the client-therapist alliance [38]. One potential validity check is for data derived from interviews to be validated against researcher analysis of the recorded session, further reducing the potential for bias in interpretive analysis.

### The research team

The two researchers are counselling and clinical psychologists who direct university programs and have held executive positions in relevant national professional bodies. They view supervision as a collaborative process grounded in the supervisory relationship and it is acknowledged that this may influence the approach to, and execution of, aspects of the study such as the recruitment and interview process and the analysis and interpretation of data. However, their professional positions and experience may also be a potential strength in terms of identifying potential experts in the field, gaining their trust and cooperation in the research, and in analysis and interpretation of the data. They will be mindful of this throughout.

### Procedure

Phase One interviews are being undertaken by either the first or second author, both experienced supervisors. Interviews are recorded on digital voice recorders and range from one to two hours. Phase 2 requires that a supervision session be DVD-recorded by the supervisor with a consenting supervisee. Then, the supervisor and supervisee are separately interviewed about the session using IPR to engage them in deeper reflections on the session [36]. These interviews are scheduled to last from one to two hours and occur 2–4 weeks after the first interview (to allow time for recording the supervision session).

### Interview method

The qualitative interview method uses open questioning to explore current understandings and experiences and make possible the development of new understandings and alternative perspectives [33].

#### Phase One

The semi-structured qualitative interview of expert supervisors includes open-ended questions covering three broad domains of practice, theory, and experience of supervision (see Table 2). These are supplemented by probes and elaborations. The in-depth interview method is used to expand and deepen the supervisors’ reflections on theory and practice, seeking to facilitate an ever-deepening reflection. Major domains explored include their approach, theory, strategies, the essence of good supervision, difficulties in supervision, repair of difficult supervisory events, and how the person of the supervisor influence their practice as a supervisor (see Table 2).

#### Phase 2

The second phase uses an IPR interview method [36] with the supervisor and supervisee separately, while reviewing a DVD of a supervision session. The researcher guides the supervisor/ee in a qualitative exploration of the dynamics of the supervision session, their understanding of what is happening, and what each is experiencing, with either researcher or interviewee able to stop and reflect on any aspect of the session. The interview involves some core questions addressing the background to the supervision session, the focus and expectations of the session, the most significant aspect of the session, how theory is applied in the session, helpful and unhelpful aspects, the quality of the supervisory relationship, and the impact of supervision on the supervisee and their practice and client outcomes (see Table 3). A wide range of probing and process questions are also asked to elaborate and deepen responses to the recorded session and gather richer data (see Table 4).

**Table 3:** Interview schedule for interviews on recorded supervision sessions

**Interview topic**	**Sample topic questions: Supervisors**	**Sample topic questions: Supervisees**
Background to supervision session	· Can you give me some background to your supervision of this supervisee?	· Can you give some background to your practice and what you are seeking from supervision?
	· How would you describe the type of supervision you are providing for this supervisee?	· How did you select your supervisor?
		· How would you describe the type of supervision you are receiving?
Focus & expectations	· What was the focus of the session?	· What was the focus of the session for you – what were you wanting?
	· What expectations did this supervisee have for the supervision?	· What expectations do you have for the supervision and how are these being met?
Most significant aspect	· What was the most significant part of the session for you? Why?	· What was the most significant part of the session for you? Why?
How theory is applied	· Can you describe how your theoretical framework is guiding your approach here?	
	· How are you applying your theory to the stage of development of this supervisee?	
Helpful aspects	· What do you think is particularly helpful in this supervision session?	· Can you give some specific examples of helpful supervision experiences? What made it helpful?
		· What do you think is particularly helpful in this supervision session?
		· What do you think you gained from the supervision session?
Unhelpful supervision	What did you do that was helpful/unhelpful in this session?	· Have you had any examples of unhelpful supervision – can you describe this?
		· What was helpful/unhelpful in this session?
Supervisory relationship	· How do you think the supervisee views you / your supervisory relationship?	· How would you describe your supervisory relationship?
Impact of supervision	· What do you think your supervisee gained from the supervision session?	· How has this session impacted on your work with this client?
	· How do you think this session may assist the supervisee in future work with their client?	· How do you think this session may assist you in future work with this client?
		· How did this influence your work with the client?

**Table 4:** Interpersonal process recall interview: Examples of prompting process questions on recorded supervision sessions

**Sample process questions in IPR interviews**	
**Supervisors**	**Supervisees**
· What prompted you to make that particular response?	· What prompted you to make that particular response?
· What were you thinking/feeling at the time?	· What were you thinking/feeling at the time?
· What was your thinking process when you asked that question/made that statement?	· What was your thinking process when you asked that question/made that statement?
· What did you make of that comment?	· What did you make of that comment?
· What did you think the supervisee was feeling when they said…?	· How would you describe the key issue for you as the supervisee here?
· How would you describe the key issue for the supervisee here?	· How would you describe the key issue for the client?
· What do you think the supervisee most needs at this point?	· What do you think you most needed at this point in the supervision?
· What might you have said/done differently?	· What might the supervisor have said/done that would have been more helpful for you?

### Analysis

The recorded interviews are being transcribed verbatim by a professional transcriber and checked for accuracy by interviewers. There will be 3 transcripts for each supervisor-supervisee dyad (a retrospective interview and an IPR interview with the DVD-recorded session for each supervisor, and an IPR interview for supervisee). The transcripts are being analyzed in three stages.

Firstly, a modified form of the consensual qualitative research method is used to derive core themes and sub-themes within domains and to determine the more prevalent themes and sub-themes (CQR) [39,40]. Analysis commences with a detailed examination of transcripts for one supervisor. This entails reading the interview transcripts a number of times and identifying emerging themes that are then categorized to create an index of domains (e.g., theories of supervision; supervisory relationship). Core themes are then identified within each domain, and sub-themes within each core theme. This recursive process is applied in multiple cycles to the remaining transcripts. Themes are progressively checked and refined by the research group until consensus is reached.

The second stage of analysis involves a form of qualitative dyadic analysis that treats the supervisory dyad as the unit of analysis. Analysis will focus on describing and comparing supervisor and supervisee reflections on their videotaped session, exploring the particular learning strategies evident in practice, comparing this with espoused theories and illuminating the reflection-in-action and reflection-on-action processes. Evidence of reflexive practices and deep learning strategies will be particularly sought. Comparative analysis will be used for supervisory dyads, by stage of supervisee development (time since training), and by supervisor theoretical orientation, to determine both common and specific factors within and between groups. The aim is to compare within-dyad perspectives on the impact of different types of interactions and supervisory processes. This will allow us to contextualise the analysis and develop an understanding of a wide range of factors influencing the *process of supervision*.

The third stage of analysis uses a theory building case study framework to guide the analysis of the supervisor-supervisee dyads in light of theories of supervision, following models established for analysing therapist-client dyads [41]. This approach emphasizes the development and refinement of theory by comparing detailed case observations to clinical research and theory [42]. Case study methodology is considered useful for research that involves a small number of information-rich participants and where the research has implications for a broad range of related phenomena [43]. Case study approaches are also considered suitable for exploratory research of inadequately understood phenomena, and where randomized control trials are impractical or unethical [44].

Theory building will occur in relation to those aspects of supervision that produce change in supervisee conceptualisation, sense of professional competence, the supervisory alliance, and impact on practice and client outcomes. Comparative analysis will also occur to determine differences between the above groups. Of particular interest will be evidence of theory/ practice tension and processes involved in trying to resolve that tension. A final more integrative stage of analysis will involve interrogating both Phase 1 and 2 data using core study variables such as theoretical orientation, common versus specific factors, and stage of development.

### Ethical issues

Ethical approval was obtained from two University Human Research Ethics Committees: La Trobe University Human Research Ethics Committee and the Curtin University Human Research Ethics Committee. Several key ethical issues are addressed in our methodology. Firstly, participants are fully informed about the study via the participant information sheet, and their written consent to participate is obtained. Secondly, supervisee participants are advised that they are free to consent and that non-consent will not affect their supervision. Thirdly, the study protects the identity of participants through use of pseudonyms and disguising any potentially identifying information. It is acknowledged that it is not possible to protect confidentiality within the supervisory dyad because participants within dyads are likely to recognise reported material provided by the other member of the dyad. However, care is taken to disguise any material that could potentially have a negative impact on the supervisory relationship. Finally, should the interviews raise any issues of concern to participants they are encouraged to raise these in their next supervision session, or alternatively to seek support through counselling.

## Discussion

Clinical supervision is widely regarded as an essential professional activity and quality assurance mechanism, not only for training new practitioners, but also to ameliorate or prevent some of the adverse impact of the work on experienced professionals [9,12]. However, there is limited research to support the claims that supervision actually has a positive impact on therapist well-being or leads to improved therapist practice and client outcomes [15]. In addition, there are only a handful of studies that point to the processes by which supervision builds, maintains, and improves therapeutic competence [45-47]. There is, therefore, considerable consensus on the need for more in-depth research into the processes of supervision and how these influence both educational and wellbeing outcomes [6,15,23].

Our study addresses some identified gaps in the research by using a qualitative mixed method design with purposive sampling of expert or highly experienced supervisors. Given the relatively early stage of the development of research on the complex process of supervision, it is important to gain a better understanding of the clinical wisdom of identified experts in the field. It is well acknowledged that when evidence is scarce, key informants or experts are a good source of data [48]. The study also adds to past research that has focused on trainees, by using supervisees who are practising professionals who have chosen their supervisor, and are in an ongoing supervisory relationship. This sampling strategy for both supervisors and supervisees increases the potential for rich data that may most efficiently shed light on the phenomenon of interest, supervisory competence.

The study is also innovative methodologically, in its focus on the supervisory dyad and use of IPR to examine both supervisor and supervisee perceptions and experiences of actual supervision sessions. Its application of a dyadic perspective in data analysis will allow for more insightful description of actual learning processes and the broader contextual factors that influence learning. A further strength of the study is the examination of the perceived impact of supervision on supervisee practice and outcomes for clients. Overall, it will provide an integrated model of supervisory practices that lead to enhanced therapeutic effectiveness.

Supervision is broadly understood to have both an educational and a support function [9]. In terms of its educational function, the study has implications for defining professional standards, designing training programs and ongoing professional development requirements, developing professional learning theory, and improving practitioner competence and client outcomes. The results will address: the nature of effective learning through supervision; how supervision learning is translated into therapist in-session practice; how this impacts on perceived client outcomes; and how supervisors can be more efficiently trained to practice supervision. The study also has the potential to cast light on how supervision may assist the integration of theory and practice, a key issue for the field [49].

Supervision also provides an important function in terms of supporting therapists and promoting wellbeing [19,47]. A better understanding of supervision processes/outcomes will inform strategies to optimise practitioner well-being. This is highly significant in a field that is characterised by high rates of professional burnout, impairment, stress, and suicide [2]. Such negative outcomes are costly, not only to the practitioner, their families and their workplaces, but to the wider society, since it takes considerable resources to train good psychotherapists. Psychotherapists deal with many of the most difficult emotional, social and traumatic problems in society, and the cumulative effect of exposure can be highly stressful. The difficult nature of their work demands a better understanding of the structural and professional support mechanisms needed to optimise performance and well-being.

## Summary and conclusion

Although supervision is core to training, ongoing professional development, and support of experienced psychotherapists, there is little research evidence regarding its efficacy and particularly its impact on therapists and clients. This study will contribute to the fledging field of supervision research by providing evidence about effective supervision practices, their impact on therapists, and the perceived impact on clients and client work. The study has the potential to provide a detailed and rich elaboration of the supervisory processes that assist to build therapist competence and confidence.

## Competing interests

The authors declare they have no competing interest.

## Authors’ contributions

Both chief investigators worked collaboratively on all aspects of design and implementation of the study. Prof Schofield took the lead role in drafting this protocol paper. All authors have read and approved the final manuscript.

## Pre-publication history

The pre-publication history for this paper can be accessed here:

http://www.biomedcentral.com/1471-244X/13/12/prepub
